# LumbriCyc: towards metabolic modelling of earthworms

**DOI:** 10.1038/s41540-026-00661-y

**Published:** 2026-02-11

**Authors:** Axel von Kamp

**Affiliations:** Analysis and Redesign of Biological Networks, Max Planck Institute for Dynamics of Complex Technical Systems, Magdeburg, Germany

**Keywords:** Computational biology and bioinformatics, Ecology, Ecology, Zoology

## Abstract

Until Charles Darwin published his book on earthworms in 1881, their role in the soil was largely unclear. By now, the importance of earthworms for soil fertility has been recognized and their phenomenology is being studied for various purposes. The investigation of earthworm molecular biology is, however, comparatively underdeveloped. This study describes the creation of BioCyc pathway genome databases for the two earthworms *Lumbricus rubellus* and *Lumbricus terrestris*, together with the first genome-scale metabolic models of these species.

## Introduction

**Fig. 1:**
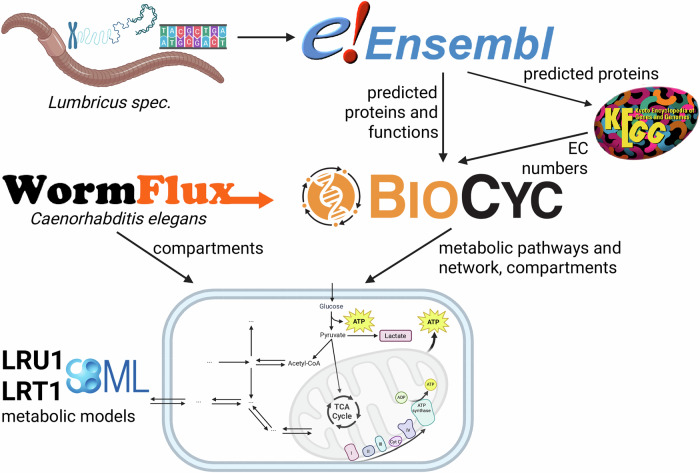
Reconstruction workflow. Overview of the steps for creating the pathway genome databases and metabolic models.

Charles Darwin extensively researched earthworms during his life and gave a comprehensive overview of their biology in his last book^1^. By now, it has been established that they are a major factor for soil fertility, and a recent meta-analysis found that, on average, earthworm presence in agroecosystems leads to a 25% increase in crop yield^2^, making them an important economic factor. Recently, the interest in earthworms has been growing^3^ and they have been used in many ecotoxicological tests^4^ for decades. In this context, their interaction with microplastics and decomposition of bioplastics has recently become a focus of attention^5^. On the molecular biology side, earthworms are being studied for their capability to regenerate body segments^6^ and for their immune system^7^. Although there is some insight into earthworm physiology^8^, no metabolic network model of any earthworm species is currently available. This study describes the creation of such models for two closely related species, *Lumbricus rubellus* and *Lumbricus terrestris*; an overview of this process is shown in Fig. 1.

For metabolic modelling of prokaryotes, it is common practice to create genome-scale metabolic models from the genome sequence, followed by some manual curation, and various tools (e.g., refs. ^9,10^) are available to support this process. For eukaryotes, like earthworms, the situation is more complicated for several reasons: Firstly, gene calling is more difficult because of the intron/exon structure of genes and the possibility of splice variants. Also, eukaryotic cells have multiple compartments, and the prediction of the cellular location of a protein from the genome sequence alone is challenging. To make matters worse, metabolite exchange between compartments can be indirect (e.g., the malate-aspartate shuttle for electron transfer between cytosol and mitochondrion) and is often not well-known. Finally, there can be cell-type-specific metabolic differences that are also difficult to predict from the genome sequence. This makes it challenging to create metabolic models for eukaryotes from the genome sequence alone. As a consequence, no universal tool for constructing compartmentalized eukaryotic metabolic models does yet exist, although there are approaches that use an existing model as a basis for reconstructing the model of another organism via the identification of orthologs (e.g., refs. ^11,12^).

Recently, the genomes of *Lumbricus rubellus*^13^ and *Lumbricus terrestris*^14^ have been sequenced by the Darwin Tree of Life project^15^. Although both species are closely related, they have different lifestyles: *L. terrestris* burrows into the soil while *L. rubellus* lives in the layer of organic litter on top of the soil. *L. terrestris* is therefore important to farming in temperate regions, whereas *L. rubellus* is, among other species, being used for vermicomposting. The latter is a process to quickly turn organic waste into compost at low temperatures (around 25 °C), whereas during microbial composting temperatures of up to 70 °C can be reached, which would be lethal to earthworms.

As the first step to create metabolic models for the two earthworm species, the annotated genomes were used to create BioCyc pathway genome databases (PGDBs)^16^ (sections “Genomic data and annotation” and “Construction of pathway genome databases”); summary statistics of the resulting databases are shown in Table 1.

**Table 1:** Genome and PGDB statistics

	Ensembl			BlastKOALA	Pathway tools	
	Genome size	Chromosomes	Predicted proteins	EC numbers	Enzymatic reactions (gap-filled)	Compounds
*L. rubellus*	1056 Mbp	18	52809	1242	2885 (229)	1981
*L. terrestris*	787 Mbp	18	37647	1232	2835 (211)	1968

Note that the number of predicted proteins includes splice variants.

From these PDGBs, metabolic models were then generated (section “Metabolic model reconstruction”). As it turns out, the PGDBs do not contain an asparagine synthase, and the models therefore lack the ability to produce this amino acid. It would be interesting to find out whether these earthworms really lack the capacity to synthesize asparagine (rendering asparagine an essential amino acid), or whether a corresponding enzyme was overlooked in the annotation process. Uniprot was searched for asparagine synthase, and the protein sequence of the most closely related organism (the leech *Helobdella robusta*) was used for a BLAST search against the predicted proteins in the PGDBs. Hits with e-values of 3e-108 and 2e-74 were found for *L. terrestris* and *L. rubellus*, respectively, and these proteins do not have a functional annotation, which makes them candidates for an asparagine synthase. Enzymes that convert asparagine to aspartate were identified for both earthworms. To enable biomass production in the models, asparagine was added as a substrate to the growth medium.

In addition, it was found that the initial *L. terrestris* model was unable to produce UMP, although the PGDB contains its biosynthesis pathway. In *L. rubellus*, the UMP biosynthesis pathway is not operational either, but there is an alternative route via uracil towards UMP that is working. The reason that UMP biosynthesis is not operational is that one of the pathway’s enzymes, the dihydroorotate dehydrogenase, is located at the inner mitochondrial membrane^17^, while the other UMP biosynthesis enzymes are located in the cytosol. Because of this seeming spatial separation, UMP biosynthesis is not operational in the model. However, it is known that the outer mitochondrial membrane is permeable for small molecules^18^. Therefore, transporters for dihydroorotate and orotate between cytosol and mitochondrial intermembrane space were added to the PGDBs, and the dihydroorotate dehydrogenase was configured to be located at the inner mitochondrial membrane and to interact with its quinone pool. With these changes, the UMP biosynthesis pathway becomes operational in both models. This example also underlines the difficulties of creating metabolic models for eukaryotes.

At this stage, the models contain some compartmentalization of their reactions, so that, for instance, the TCA cycle is located in the mitochondrion and the respiratory chain in its inner membrane. Most of the reactions are by default placed in the cytosol, and there are no transporters between the compartments, which means that, for instance, the TCA cycle and respiratory chain cannot operate because they are not connected to metabolites of the cytosol. To additionally assign compartments to the metabolites and reactions, a semi-automatic procedure was developed to use an existing *C. elegans* model (iCEL1314^19^) as a template (section “Augmenting compartmentalization”). Importantly, transporters between the cytosol and mitochondrion are added at this stage.

Some model curation (section “Model refinement”) was performed, and as a major result, compartmentalized models that support growth are derived for both species. The compartments cytosol, mitochondrial intermembrane space, mitochondrial lumen, and extracellular space are distinguished in the models.

The earthworm models have a working respiratory chain with a P/O ratio of 2.5. Complete oxidation of one molecule of glucose would generate 34 ATP, while in the iCEL1314 model, it would generate 46 ATP. The latter value is unrealistically high and indicates that there are modelling issues with the energy metabolism in the iCEL1314 model. If a template-based model reconstruction method had been used (e.g., refs. ^11,12^.), then this issue would also have been carried over into the earthworm models.

Furthermore, the *Lumbricus* models reproduce certain characteristics of earthworm physiology: It is known that these organisms can survive without oxygen for up to one day and in such a situation release various fermentation products (lactate, succinate, alanine, acetate, and propionate) into their environment^20^. The *Lumbricus* models can produce all these fermentation products. In addition, cellulases^21^ and chitinases^22^ have been found in some earthworm species, and corresponding enzymatic reactions are present in both *Lumbricus* PGDBs. Both models can metabolize the monomers (β-glucose/N-acetyl-D-glucosamine) that are produced by cellulases/chitinases, respectively.

Table 2 shows the number of model components of the newly created *Lumbricus* models. Compared to the PGDBs, there are significantly fewer metabolites and reactions in the model. The main reason for this is a technical one, in that MetaFlux does not export reactions that can be derived to be inoperative by inspecting the network structure (e.g., reactions that involve dead-end metabolites). Consequently, there are somewhat fewer metabolites and reactions in the *Lumbricus* models than in iCEL1314, but also fewer blocked reactions because many blocked reactions present in the PGDBs will not be part of the models. Also note, that the blocked reactions were computed with the default medium of the respective model, which contains many more different nutrients in the iCEL1314 model compared to the *Lumbricus* models. When looking at the high number of genes in the *Lumbricus* models, it has to be considered that many alternative splice variants are predicted by the Ensembl annotation pipeline. Whether all these variants actually occur in the cells is unclear in the absence of transcriptomics data.

**Table 2:** Basic information on the two *Lumbricus* models created here in comparison to the iCEL1314 model

Model name (species)	Compartments	Metabolites	Reactions (blocked), gap-filled, mitochondrial	Genes
LRU1 (*L. rubellus*)	c, m, i, e	1468	1751 (679), 73, 159	2334
LRT1 (*L. terrestris*)	c, m, i, e	1486	1819 (689), 72, 160	2133
iCEL1314 (*C. elegans*)	c, m, e	1533	2230 (866), 101, 277	1314

The model compartments are: c: cytosol, m: mitochondrion, i: mitochondrial intermembrane space, e: extracellular. A reaction is considered to be blocked if it cannot carry any flux when only biomass production with the default medium of the respective model is enabled. Therefore, if additional metabolites were added to the media or additional excretion products allowed, further reactions can become unblocked.

The developed models provide a useful resource to start a systematic analysis of the metabolism of earthworms. Unfortunately, there is currently a lack of validation data to further curate the models. Although earthworm toxicogenomics is an active research area^23^, the data available are mostly not directly suitable for metabolic model validation, which would ideally require flux measurements or gene essentiality data. Besides model validation, when suitable data becomes available, possible further work includes the specialization of the models into cell-type specific variants, using scRNA data, to better understand the earthworm organism as a whole. Furthermore, the analysis of the earthworm microbiomes with meta-genomics and meta-proteomics techniques should make it possible to better understand the ecological role of the involved organisms.

## Methods

### Genomic data and annotation

The genome sequences for *Lumbricus rubellus* and *Lumbricus terrestris* were obtained from the Darwin Tree of Life project^15^, which uses the Ensembl database^24^ as data storage. Primary annotation, including gene calling and functional annotation, was derived from the Ensembl automated annotation pipeline. To augment this, the proteins predicted by Ensembl were subjected to additional annotation using KEGG’s BlastKOALA^25^ tool, from which Enzyme Commission (EC) number assignments were extracted.

### Construction of pathway genome databases (PGDBs)

The combined data from Ensembl and BlastKOALA were processed to generate input files for Pathway Tools^26^ software (version 29.0). The PathoLogic component of this software was used to create BioCyc PGDBs for both earthworm species. This process involves adding whole pathways from the MetaCyc reference database to the PGDBs, depending on a scoring scheme that evaluates how many reactions of a pathway are (exclusively) found in the annotated genome. This can be seen as a gap-filling procedure without the explicit use of a biomass objective.

### Metabolic model reconstruction

Genome-scale metabolic models^27^ were generated from the PGDBs using MetaFlux^28^, a constraint-based modelling component of Pathway Tools. The model setup included the following configuration:

- *Biomass composition*: for creating a metabolic model, MetaFlux integrates a default biomass reaction into the model to check whether it supports growth. This biomass composition is generic to all cellular organisms and mainly comprises amino acids, DNA, as well as RNA components. Because no lipids are present in this generic biomass, some fatty acids were added for the creation of the earthworm models to make them more biologically realistic.
- *Maintenance energy*: MetaFlux itself does not add any growth or non-growth-associated ATP consumption to the model. Actual values for these are not known and could also be cell-type-specific, but a growth-associated ATP consumption of 20 was added to the model, which should be seen as a lower limit.
- *Growth medium*: as substrate, a mixture of glucose, amino acids essential for metazoans, and several vitamins is used in the models. This contrasts with most microorganism models, which are typically set up to simulate growth on a minimal medium, something that would not be possible for animal cells.

Initially, the models did not produce biomass and were manually curated to address specific metabolic gaps:

- *Asparagine biosynthesis*: asparagine was added to the growth medium because no asparagine synthase was present in the PGDBs.
- *UMP biosynthesis*: to fix a non-operational UMP pathway caused by compartment separation, the enzyme dihydroorotate dehydrogenase was configured in the PGDBs to localize at the inner mitochondrial membrane and to interact with the membrane’s quinone pool. Additionally, transporters for orotate and dihydroorotate were added to the PGDBs to allow their exchange between the cytosol and the mitochondrial intermembrane space. These transporters are technical reactions only because, in reality, the outer mitochondrial membrane is permeable for small molecules^18^.

### Augmenting compartmentalization

MetaFlux was used to export the metabolic models in SBML format^29^. At this stage, the models contain some compartmentalization of their reactions, so that, for instance, the TCA cycle is located in the mitochondrion and the respiratory chain in its inner membrane. Most of the reactions are by default placed in the cytosol, and there are no transporters between the compartments, which means that, for instance, the TCA cycle and respiratory chain cannot operate because they are not connected to metabolites of the cytosol. To additionally assign compartments to the metabolites and reactions, a semi-automatic procedure was developed to use an existing *C. elegans* model (iCEL1314^19^) as a template, which comprises cytosol, mitochondrion, and an extracellular compartment. This required the following steps:

- *Metabolite mapping*: iCEL1314 uses the biochemistry from BiGG^30^, whereas the BioCyc PGDBs have their own biochemistry. Their metabolite and reaction identifiers are distinct, and the same metabolite can be protonated differently in these biochemistries, leading to different formulas and charges. Although BioCyc provides links to BiGG for many metabolites, this is not a complete mapping, and additional relations between BioCyc and BiGG metabolites were derived with the help of MetaNetX^31^. Further relations were derived via KEGG identifiers in the annotation of the BioCyc and iCEL1314 metabolites. For the mappings, it is then checked that the metabolites have the same formula and charge, except for discrepancies stemming from pronation differences, which are allowed.
- *Reaction mapping*: if all metabolites of an earthworm model reaction can be mapped to iCEL1314 metabolites, it is checked whether it is present in that model. Again, differences between proton balances are allowed for mapping purposes, but the reactions themselves always remain mass- and charge-balanced.
- *Compartmentalization*: if an earthworm model reaction (that is not explicitly designated as mitochondrial) can be mapped to an iCEL1314 reaction, the compartment assignment for this reaction is taken from the iCEL1314 model. Additionally, some reactions are assigned to compartments manually, where a mapping to the iCEL1314 model could not be established. For instance, fatty acid synthesis in iCEL1314 is modeled without the acyl-carrier proteins, but these are made explicit in the BioCyc models, so that for these reactions an automatic mapping between both models was not possible.
- *Transport reactions between mitochondrion and cytosol*: The iCEL1314 model contains a set of transporters between the mitochondrion and the cytosol. If the participating metabolites of such a transporter are present in the earthworm model, then this transporter is incorporated into this model.

There are several manual additions and corrections required at various stages of this process to improve the mapping between the models; these can be found in the provided scripts.

### Model refinement

The reversibility of several reactions was modified to increase the biological realism of the model: All decarboxylases were made irreversible to prevent unrealistic CO_2_-fixation, and isomerases were made reversible. A dGDP hydrolase (RXN-14218) and an ATP hydrolase (RXN_10862) were made irreversible to prevent free ATP regeneration. Also, the phosphate (PIt2m) and pyruvate (PYRt2m) transporters between the cytosol and the mitochondrion were set up as proton symporters, which are the typical mitochondrial transporters in eukaryotic models that are listed in the BiGG database.

## Data Availability

The PGDBs can be downloaded from Zenodo (10.5281/zenodo.16021633) or via the Pathway Tools Registry. The PGDBs can also be accessed at https://lumbricyc.mpi-magdeburg.mpg.de/. The scripts for creating Pathway Tools input and the metabolic models are available on GitHub (https://github.com/cnapy-org/earthworm-models) together with the models themselves.

## References

[1] Darwin, C. *The Formation of Vegetable Mould, Through the Action of Worms, with Observations on Their Habits* (John Murray, 1881).

[2] Groenigen, J. W. et al. Earthworms increase plant production: a meta-analysis. *Sci. Rep.* **4**, 6365 (2014). 25219785 10.1038/srep06365 PMC5376159

[3] Stürzenbaum, S. R., Andre, J., Kille, P. & Morgan, A. J. Earthworm genomes, genes and proteins: the (re)discovery of Darwin’s worms. *Proc. Biol. Sci.* **276**, 789–797 (2009). 19129111 10.1098/rspb.2008.1510 PMC2664377

[4] Peijnenburg, W. J. G. M. & Vijver, M. G. Earthworms and their use in eco(toxico)logical modeling. in *Ecotoxicology Modeling* (ed. Devillers, J.) 177–204 (Springer US, 2009).

[5] Holzinger, A. et al. Biodegradable polymers boost reproduction in the earthworm Eisenia fetida. *Sci. Total Environ.* **892**, 164670 (2023). 37290643 10.1016/j.scitotenv.2023.164670

[6] Shao, Y. et al. Genome and single-cell RNA-sequencing of the earthworm Eisenia andrei identifies cellular mechanisms underlying regeneration. *Nat. Commun.* **11**, 2656 (2020). 32461609 10.1038/s41467-020-16454-8 PMC7253469

[7] Homa, J. Earthworm coelomocyte extracellular traps: structural and functional similarities with neutrophil NETs. *Cell Tissue Res.* **371**, 407 (2018). 29404728 10.1007/s00441-018-2787-0 PMC5820388

[8] Edwards, C. A. & Arancon, N. Q. Earthworm physiology. in *Biology and Ecology of Earthworms* (eds. Edwards, C. A. & Arancon, N. Q.) 33–54 (Springer US, 2022).

[9] Seaver, S. M. D. et al. The ModelSEED Biochemistry Database for the integration of metabolic annotations and the reconstruction, comparison and analysis of metabolic models for plants, fungi and microbes. *Nucleic Acids Res.* **49**, D575–D588 (2021). 32986834 10.1093/nar/gkaa746 PMC7778927

[10] Machado, D., Andrejev, S., Tramontano, M. & Patil, K. R. Fast automated reconstruction of genome-scale metabolic models for microbial species and communities. *Nucleic Acids Res.* **46**, 7542–7553 (2018). 30192979 10.1093/nar/gky537 PMC6125623

[11] Capela, J. et al. merlin, an improved framework for the reconstruction of high-quality genome-scale metabolic models. *Nucleic Acids Res.* **50**, 6052–6066 (2022). 35694833 10.1093/nar/gkac459 PMC9226533

[12] Han, W. et al. AlphaGEM enables precise genome-scale metabolic modelling by integrating protein structure alignment with deep-learning-based dark metabolism mining. Preprint at 10.1101/2025.07.21.665674 (2025). 10.1038/s41467-026-75549-w PMC13490510 42463708

[13] Short, S. et al. The genome sequence of the red compost earthworm, Lumbricus rubellus (Hoffmeister, 1843). *Wellcome Open Res.* **8**, 354 (2023). 38618197 10.12688/wellcomeopenres.19834.1 PMC11015115

[14] Blaxter, M. L. et al. The genome sequence of the common earthworm, Lumbricus terrestris (Linnaeus, 1758). *Wellcome Open Res.* **8**, 500 (2023). 38249959 10.12688/wellcomeopenres.20178.1 PMC10799228

[15] Darwin Tree of Life. https://projects.ensembl.org/darwin-tree-of-life/.

[16] Karp, P. D. et al. The BioCyc collection of microbial genomes and metabolic pathways. *Brief. Bioinform.* **20**, 1085–1093 (2019). 29447345 10.1093/bib/bbx085 PMC6781571

[17] Rawls, J., Knecht, W., Diekert, K., Lill, R. & Löffler, M. Requirements for the mitochondrial import and localization of dihydroorotate dehydrogenase. *Eur. J. Biochem.* **267**, 2079–2087 (2000). 10727948 10.1046/j.1432-1327.2000.01213.x

[18] Alberts, B. et al. The mitochondrion. in *Molecular Biology of the Cell*. 4th edn (Garland Science, 2002).

[19] Yilmaz, L. S. et al. Modeling tissue-relevant Caenorhabditis elegans metabolism at network, pathway, reaction, and metabolite levels. *Mol. Syst. Biol.* **16**, e9649 (2020). 33022146 10.15252/msb.20209649 PMC7537831

[20] Gruner, B. & Zebe, E. Studies on the anaerobic metabolism of earthworms. *Comp. Biochem. Physiol. Part B Comp. Biochem.* **60**, 441–445 (1978).

[21] Ueda, M. et al. Cloning and expression of the cold-adapted endo-1,4-β-glucanase gene from Eisenia fetida. *Carbohydr. Polym.* **101**, 511–516 (2014). 24299806 10.1016/j.carbpol.2013.09.057

[22] Kim, D. et al. A novel chitinase from the earthworm, Eisenia andrei. *Anim. Cells Syst.* **20**, 48–51 (2016).

[23] Gong, P. & Perkins, E. J. Earthworm toxicogenomics: A renewed genome-wide quest for novel biomarkers and mechanistic insights. *Appl. Soil Ecol.* **104**, 12–24 (2016).

[24] Dyer, S. C. et al. Ensembl 2025. *Nucleic Acids Res.* **53**, D948–D957 (2025). 39656687 10.1093/nar/gkae1071 PMC11701638

[25] Kanehisa, M., Sato, Y. & Morishima, K. BlastKOALA and GhostKOALA: KEGG tools for functional characterization of genome and metagenome sequences. *J. Mol. Biol.* **428**, 726–731 (2016). 26585406 10.1016/j.jmb.2015.11.006

[26] Karp, P. D. et al. Pathway Tools version 23.0 update: software for pathway/genome informatics and systems biology. *Brief. Bioinform.* **22**, 109–126 (2021). 31813964 10.1093/bib/bbz104 PMC8453236

[27] Bordbar, A., Monk, J. M., King, Z. A. & Palsson, B. O. Constraint-based models predict metabolic and associated cellular functions. *Nat. Rev. Genet.* **15**, 107–120 (2014). 24430943 10.1038/nrg3643

[28] Latendresse, M., Ong, W. K. & Karp, P. D. Metabolic modeling with MetaFlux. *Methods Mol. Biol.* **2349**, 259–289 (2022). 34718999 10.1007/978-1-0716-1585-0_12

[29] Hucka, M. et al. The systems biology markup language (SBML): a medium for representation and exchange of biochemical network models. *Bioinformatics* **19**, 524–531 (2003). 12611808 10.1093/bioinformatics/btg015

[30] King, Z. A. et al. BiGG Models: a platform for integrating, standardizing and sharing genome-scale models. *Nucleic Acids Res.* **44**, D515–D522 (2016). 26476456 10.1093/nar/gkv1049 PMC4702785

[31] Moretti, S., Tran, V., Mehl, F., Ibberson, M. & Pagni, M. MetaNetX/MNXref: unified namespace for metabolites and biochemical reactions in the context of metabolic models. *Nucleic Acids Res.* **49**, D570–D574 (2021). 33156326 10.1093/nar/gkaa992 PMC7778905

